# OXPHOS Supercomplexes as a Hallmark of the Mitochondrial Phenotype of Adipogenic Differentiated Human MSCs

**DOI:** 10.1371/journal.pone.0035160

**Published:** 2012-04-16

**Authors:** Andreas D. Hofmann, Mandy Beyer, Udo Krause-Buchholz, Manja Wobus, Martin Bornhäuser, Gerhard Rödel

**Affiliations:** 1 Institute of Genetics, Technical University of Dresden, Dresden, Germany; 2 Medical Clinic and Polyclinic I, University Hospital ‘Carl Gustav Carus’ Technical University of Dresden, Dresden, Germany; RWTH Aachen University Medical School, Germany

## Abstract

Mitochondria are essential organelles with multiple functions, especially in energy metabolism. Recently, an increasing number of data has highlighted the role of mitochondria for cellular differentiation processes. Metabolic differences between stem cells and mature derivatives require an adaptation of mitochondrial function during differentiation. In this study we investigated alterations of the mitochondrial phenotype of human mesenchymal stem cells undergoing adipogenic differentiation. Maturation of adipocytes is accompanied by mitochondrial biogenesis and an increase of oxidative metabolism. Adaptation of the mt phenotype during differentiation is reflected by changes in the distribution of the mitochondrial network as well as marked alterations of gene expression and organization of the oxidative phosphorylation system (OXPHOS). Distinct differences in the supramolecular organization forms of cytochrome *c* oxidase (COX) were detected using 2D blue native (BN)-PAGE analysis. Most remarkably we observed a significant increase in the abundance of OXPHOS supercomplexes in mitochondria, emphasizing the change of the mitochondrial phenotype during adipogenic differentiation.

## Introduction

Mitochondria are the site of the oxidative phosphorylation system (OXPHOS) that comprises four enzyme complexes of the respiratory chain (RC), two mobile electron carriers (ubiquinone, cytochrome *c*) as well as the ATP synthase. Electrons derived from energy-rich carbohydrates are transported from the citric acid cycle via NADH and succinate to complex I (CI) or complex II (CII), respectively. Ubiquinone and cytochrome *c* are mediating the further transport of electrons to complex III (CIII) and complex IV (CIV). During the redox reactions at CI, CIII and CIV protons are transferred through the inner mitochondrial (mt) membrane leading to an electrochemical gradient that is utilized by the ATP synthase (CV; complex V) to produce ATP. The protein complexes of the OXPHOS system are encoded by two genomes requiring a coordinated synthesis and assembly into functional entities to establish an active RC.

Beside their well-known function in energy supply, mitochondria play pivotal roles in other essential cellular processes, such as the formation of Fe-S-clusters, calcium homeostasis, oxygen sensing, cellular proliferation, apoptosis and aging [1]. By now it is becoming evident that mitochondria are also important for cell differentiation processes [2], [3], [4]. For instance, it was shown that mt biogenesis accompanies differentiation of 3T3-L1 fibroblast cell lines into brown adipocytes [5]. The peroxisome proliferator-activated receptor gamma coactivator 1-alpha (PGC-1α) that acts as a transcriptional coactivator, was identified as the key regulator of mt biogenesis [6].

Due to the very distinct metabolic and energetic demands of differentiated cells types, stem cell differentiation not only requires mt biogenesis, but in addition a specific adaptation of the mt function [7], [8], [9]. Fully differentiated cells are characterized by the size of the mt compartment, the copy number of mtDNA, and the intracellular localization and morphology of mitochondria, subsumed as a specific mt phenotype [10]. Differences regarding the mt phenotype between stem cells and differentiated cells were reported recently [2].

Undifferentiated cells are housed and maintained in stem cell niches, *e.g.* human mesenchymal stem cells/mesenchymal stromal cells with stem cell capacity (hMSCs) within certain areas of the bone marrow. Their energy supply is more dependent on glycolysis or alternative anaerobic metabolism than on OXPHOS [2], [11]. Main reasons are (i) the low oxygen concentrations in the stem cell niches, *e.g.* 0–4% O_2_ in the bone marrow [12]; (ii) the low proliferative activity of stem cells; and (iii) the avoidance of reactive oxygen species (ROS) formation [1], [13]. In line with low OXPHOS and high glycolytic activity ATSCs (a form of a primate adult stromal cell line), human hematopoietic stem cells (HSCs) and mouse embryonic stem cells (ESCs) have been shown to possess few, mainly perinuclear localized mitochondria [11], [14].

Upon initiation of and during differentiation, a metabolic switch towards OXPHOS is necessary to meet the cells' energy demand, thereby requiring mt biogenesis [3]. OXPHOS activity, as a key feature of the mt phenotype, requires the coordinated synthesis of mt and nuclearly encoded proteins and their assembly in the OXPHOS enzyme complexes. OXPHOS complexes have been reported to be organized in supramolecular structures, so called supercomplexes (SCs) or respirasomes [15], [16]. The formation of SCs is supposed to enhance electron transport chain (ETC) efficiency and maybe linked to cellular differentiation state and diseases [17], [18]. Only few data are available on the mt phenotype of primary cells such as hMSCs in the course of their differentiation [19]. In this study we address the role of mitochondria in stem cell differentiation by investigating the mt phenotype of hMSCs in the course of adipogenic differentiation, with special emphasis on the molecular organization of OXPHOS complexes. Our data show that adipogenic differentiation of hMSCs is accompanied by the increased formation of OXPHOS supercomplexes.

## Materials and Methods

### Ethics Statement

hMSCs were obtained from bone marrow aspirates of healthy donors after informed consent, both written and oral. The collection and the usage of hMSCs for research issues was approved by a vote of the ethics committee of the Technische Universität Dresden (IRB approval no. EK263122004) (Ethikkommission der TU Dresden, http://www.ek.med.tu-dresden.de/). All data were analyzed anonymously.

### Isolation of bone marrow mononuclear cells and culture of hMSCs

MSCs were isolated as described recently [20]. Briefly, after a 1∶5 dilution with PBS (Invitrogen, Karlsruhe Germany) the mononuclear cell fraction was isolated by density gradient centrifugation at 900× g for 30 min at RT using Biocoll solution (Biochrom, Berlin Germany; density 1,077 g/l) and seeded at a density of 6×10^4^ to 1×10^5^/cm^2^ (P0) into T175 cell culture flasks (Greiner, Frickenhausen Germany) in DMEM containing 10% FBS. Medium was first changed within 2 days after isolation to remove non adherent cells and afterwards twice a week. Cells were harvested at subconfluence using TrypZean (Sigma Aldrich, Steinheim, Germany; GMP grade equivalent of trypsin). Cells at passage 1 and thereafter were plated at a mean density of 5×10^3^/cm^2^.

### Cell culture

hMSCs were maintained in DMEM containing 1 g/L Glucose, 10% fetal bovine serum and 2 mM Glutamine (37°C, 5% CO_2_). Cells were passaged when the culture dishes were confluent. Medium was changed twice per week. Cells were harvested by trypsin treatment (1× Trypsin-EDTA (PAA), 5 min at 37°C). hMSCs were washed, pelleted and stored at −80°C. All experiments were performed with cells from the second passage (P2). For protein analysis hMSCs from up to six different donors with a balanced gender distribution were pooled.

### Adipogenic differentiation of hMSCs

Adipogenic differentiation of hMSCs was induced in 80% confluent cultures (P2) as described previously by Pittenger *et al.* [19]. Differentiation medium was changed twice per week. Adipogenic differentiated cells were harvested on day 14 after induction of differentiation by trypsin treatment (1× Trypsin-EDTA (PAA), 5 min at 37°C). Cells were washed, pelleted and stored at −80°C.

### Microscopy

Brightfield micrographs were taken using the inverted microscope Axio Observer.Z1 (Carl Zeiss AG, Jena) with a 10×/0.30 Ph1 objective and AxioCam MRm (Carl Zeiss AG, Jena). For Oil Red O staining of lipid droplets a stock solution (2 mg/ml) of Oil Red O (Sigma-Aldrich, St. Louis) was prepared in 99% ethanol. Cells were fixed in 10% paraformaldehyde for 30 min and stained with freshly prepared staining solution (70% filtered Oil Red O stock solution in ddH_2_O) for 25 min at room temperature. Subsequently, cells were rinsed with ddH_2_O (10 sec.) and washed with 60% isopropanol for 5 min. For fluorescence microscopy (FM) the nuclei were stained blue by incubating the cells for 5 min at 37°C with DAPI (AppliChem) at a final concentration of 8 µg/ml, mitochondria were stained green by incubating cells in medium containing 50 nM MitoTracker® Green FM (Invitrogen) for 2 min at 37°C. Plasma membrane was stained orange by incubating cells in medium containing 10 µg/ml CellMask™ orange plasma membrane stain (Invitrogen) for 6 min at 37°C. Pictures were taken using a Keyence Biozero microscope and a Plan Apo 100× (VC 100×/ 1.40 Oil) objective (Nikon).

### Gene expression analysis

A custom version of the StellARray qPCR Array (Lonza) was employed following the manufacturer's protocol. Briefly, total RNA was isolated from cell pellets using the RNeasy® kit (Qiagen), and subsequently DNA was digested on column using DNase I (High Pure RNA Isolation Kit, Roche) for 30 min at room temperature. RNA quality was validated using a Bioanalyzer in combination with the RNA 6000 Nano Kit (Agilent Technologies) and quantified with a NanoDrop ND-1000 Spectrophotometer (Nanodrop Technologies). 500 ng of total RNA was used for cDNA synthesis using the ‘Revert Aid First Strand cDNA synthesis kit®’ (Fermentas) and random hexameric primers. A 1∶10 dilution of the cDNA was used for the qPCR using a 96-well format of the custom designed StellARray. qPCR was executed using SYBR Green PCR (2×) Master Mix (Fermentas) and a Master Cycler gradient® (Eppendorf). qPCR data were analyzed using the Global Pattern Recognition™ (GPR) data analysis tool (Lonza) and represent multiple biological replicates: hMSCs (n = 6) and adipocytes (n = 4).

### Isolation of mitochondria

Cells (hMSCs and adipocytes) were harvested by trypsin treatment. Mitochondria of confluent hMSCs and of adipocytes were isolated using the ‘Mitoprofile® Mitochondria Isolation Kit For Cultured Cells with Homogenizer’ (MitoSciences/Invitrogen) following the instructions of the manufacturer. Mitochondrial pellets were resuspended in isolation buffer (250 mM sucrose, 30 mM MOPS, pH 7.2), shock frozen in liquid nitrogen and stored at −80°C.

### 2D Blue native/ SDS polyacrylamide gel electrophoresis

Blue native (BN) PAGE was performed basically as described elsewhere [16], [21] using polyacrylamide gradient gels (3–13%), electrophoresis was carried out on ice over night. For the 2D-SDS-PAGE, stripes from the first dimension gel were sliced and proteins were denatured by incubation in 5 mM TCEP and 1% SDS (w/v) for 15 min at RT. Electrophoretic separation in the second dimension was carried out as described elsewhere [21].

### Western blot analysis

Electrophoresis and blotting of 2D gels was performed essentially as described [22]. Primary antibodies were directed either against complex CIV (subunit COX1, 57 kDa) (Invitrogen) or against complex I (subunit ND6, 20 kDa), complex II (subunit FeS, 30 kDa), complex III (subunit core 2, 47 kDa), complex IV (subunit COX2, 26 kDa), and complex V (subunit α, 55 kDa), which are components of the Anti-Hu Total OxPhos Complex Kit an antibody-cocktail (Invitrogen).

## Results

### Differentiation of hMSCs into adipocytes results in changes of the mt network

Adipogenic differentiation of hMSCs was induced as described previously by Pittenger *et al.* [19]. After three days tiny lipid droplets became visible by standard light microscopy, even in the absence of specific fat staining. Onset of differentiation was not completely synchronized, and 10–40% of the cells did not differentiate at all. This can be explained by the fact that primary hMSCs, isolated from bone marrow, represent a relatively heterogeneous population that contains already committed cells like ‘preadipocytes’ and ‘preosteoblasts’ beside hMSCs.

**Figure 1:**
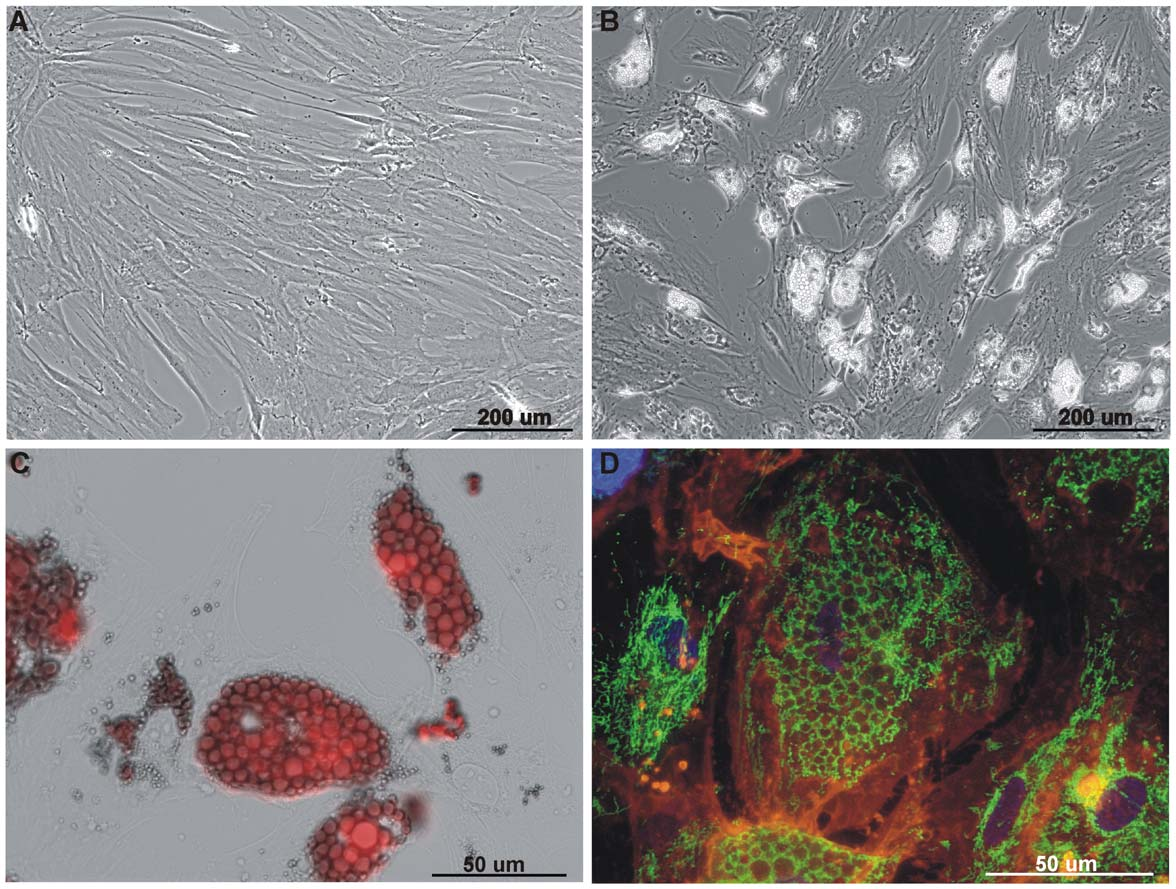
Mitochondrial network of adipogenic differentiated hMSCs. All pictures show cells of the second passage (P2). Representative brightfield LM-micrographs of (A) undifferentiated hMSCs, (B) adipocytes at day 14 after induction of differentiation (100×), and (C) Oil Red O stained adipocytes 14 days after induction of differentiation (400×). (D) Representative FM-micrograph (overlay) of an adipogenic differentiated hMSC culture 14 days after induction of differentiation (500×). In the center an adipocyte with multiple lipid droplets is seen. Nuclei stained with DAPI (blue), mt network stained with MitoTracker Green FM (green), and plasma membrane stained with CellMask Orange (orange).

The formation of multilocular lipid droplets proves adipocyte differentiation. In differentiating cells both the number and the size of lipid droplets increased until days 14–21, when they fused to form less but bigger lipid droplets (Figure 1A and 1B). Lipid droplets were clearly documented by Oil Red O staining (Figure 1C).

In line with the observations in a primate stem cell line [23] and hHSCs [24] fluorescence microscopy revealed clear differences in the morphology of the mt network between undifferentiated and differentiated cells (Figure 1D): while undifferentiated cells mainly show a perinuclear clustering of mitochondria ([23] and own observations), mitochondria of adipocytes are more evenly distributed in the cytoplasm, preferentially around the lipid droplets. This specific distribution of the mt network was observed in all adipocytes analyzed.

### Gene expression analysis during adipogenic differentiation indicates mt biogenesis and enhanced oxidative metabolism

**Figure 2:**
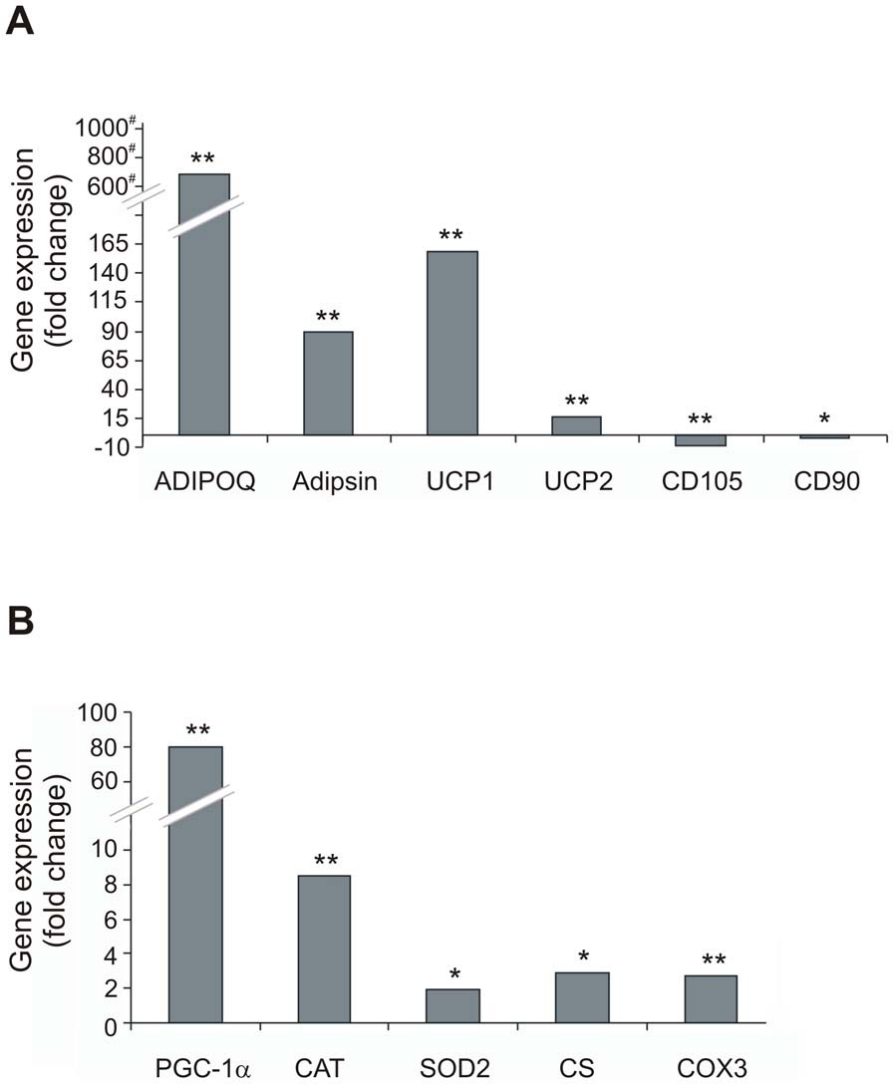
Gene expression during adipogenic differentiation of hMSCs. mRNA levels of selected genes were determined by StellARray analysis in adipocytes and undifferentiated hMSCs. The respective ratios are given as x-fold change, # ×1000. (A) Analysis of selected hMSC and differentiation markers: CD105 and CD90 are hMSC marker genes, ADIPOQ, adipsin and uncoupling protein 1 and 2 (UCP1, UCP2) are marker genes for adipocytes. (B) Analysis of selected mt biogenesis marker and genes involved in oxidative metabolism: PGC-1α, catalase (CAT), superoxide dismutase (SOD2), citrate synthase (CS) and subunit COX 3. All data are statistically significant: *p<0.05, **p<0.001.

We employed a custom designed array containing genes involved in mt biogenesis, OXPHOS, and hMSC differentiation. 14 days after initiation of differentiation the mRNA levels of all tested adipogenic marker proteins were statistically significant increased: Adipsin, UCP1 and UCP2 showed a 16 to 160-fold increase, and ADIPOQ expression was even enhanced by a factor of ∼650.000 when compared to hMSCs (Figure 2A). In parallel, mRNA levels of hMSC marker proteins CD105 and CD90 were decreased by factors of ∼9 and 3, respectively (Figure 2A). Adipsin is a general adipocyte marker and ADIPOQ a marker of white adipocyte tissue, whereas other adipogenic marker proteins such as uncoupling protein 1 (UCP1) and uncoupling protein 2 (UCP2) are known to be exclusively or predominantly expressed in brown adipocyte tissue. These data confirm the adipogenic cell fate that is accompanied by the morphological changes as described above. To assess mt biogenesis we included PGC-1α known as the master regulator of mt biogenesis. The mRNA levels of PGC-1α were statistically significant increased in adipocytes by a fold change factor of 80, when compared to hMSCs (Figure 2B). Representative genes involved in oxidative metabolism showing a statistically significant regulation, namely catalase (CAT), mt manganese superoxide dismutase 2 (SOD2), citrate synthase (CS) and subunit 3 of cytochrome *c* oxidase (COX3) were chosen to demonstrate the metabolic switch. During adipogenic differentiation the mRNA levels of COX3, CS were 3-fold, SOD2 2-fold and CAT 9-fold increased (Figure 2B). A similar increase in the expression of genes indicating the metabolic switch and mt biogenesis was observed during osteogenic differentiation (data not shown). CAT and SOD2 are ROS detoxifying enzymes with an important protective function during oxidative metabolism. CS, an essential enzyme of the Krebs tricarboxylic acid cycle and the mt encoded COX3 as an essential catalytic subunit of cytochrome *c* oxidase are prominent indicators for a metabolic switch towards OXPHOS during adipogenic differentiation.

### Organization of OXPHOS complexes changes during adipogenic differentiation of hMSCs

To uncover differences in the OXPHOS organization between hMSCs and their differentiated derivatives such as adipocytes, the organization of the five OXPHOS complexes was studied by 2D-BN/SDS-PAGE and a subsequent Western blot (WB) analysis using antibodies against individual subunits from each complex. The specific WB signal pattern representing the organization of OXPHOS complexes is dubbed as ‘OXPHOS profile’.

Mitochondria were isolated from undifferentiated hMSCs and adipocytes and solubilized with the mild detergent digitonin, which preserves the supramolecular organization of OXPHOS complexes [15]. To address whether normoxic culture conditions (∼21% O_2_) *per se* affect the supramolecular organization of the OXPHOS, hMSCs that were cultivated without differentiation for 14 days served as a control.

**Figure 3:**
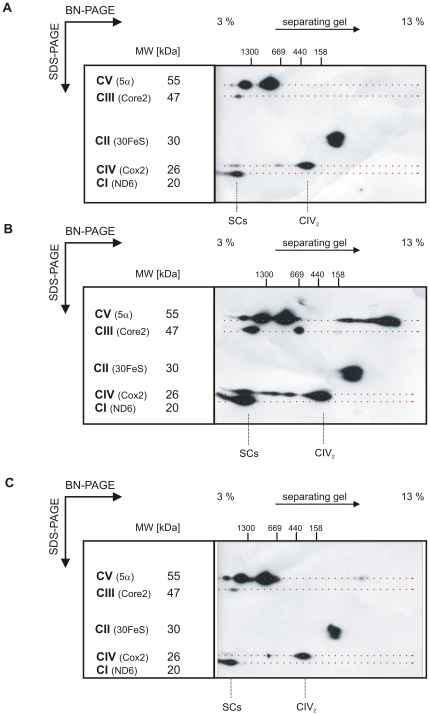
Supramolecular organization of OXPHOS complexes. Identical amounts of mt proteins from undifferentiated hMSCs (A), adipocytes (B), and undifferentiated hMSCs cultivated for 14 days (control, C) were separated on a 2D-BN/SDS-PAGE, blotted and detected with antibodies directed against individual subunits of all OXPHOS complexes. Molecular weight as well as the supercomplexes (SCs) and the COX dimer (CIV_2_, MW 400 kDa) are indicated. The regions of SCs comprises forms a, b, c and d according to Schägger and Pfeiffer (2000). Dotted lines indicate assembly forms of the respective annotated respiratory chain complex.

The OXPHOS profile of adipocytes clearly differed from that of hMSCs (Figure 3B and 3A, respectively). Adipocytes possess a typical OXPHOS complex organization as present in many terminally differentiated cells, e.g. HeLa cells or PBMCs [18], [21]. In contrast, the OXPHOS profile of hMSCs contains less and generally weaker signals of the OXPHOS complexes: the dimeric form of CIII that is present in adipocyte mitochondria, is neither detectable in mitochondria of hMSC nor of the control cells. Moreover, stem cells exhibit a reduced number and a fainter signal intensity of supramolecular CIV assembly forms (as revealed by the COX2 signal) compared to differentiated cells. Most remarkably, however, the signals for the various forms of OXPHOS SCs are much stronger pronounced in adipocytes compared to undifferentiated cells. The area of SCs encompasses the major SC (∼1.7 MDa) consisting of one CI, a CIII dimer and one CIV (CI CIII_2_ CIV) and in addition CI, CIII_2_ assemblies with no or more than one CIV entities [25]. Prolonged culture of hMSCs under normoxic conditions without induction of differentiation does not lead to such a prominent occurrence of SCs as observed in adipocytes (Figure 3C). Noteworthy is the occurrence of free CV subunit α (5α) and its assembly intermediates at a molecular range between 55 and 160 kDa as well as an increase of the CI signal exclusively observed in adipocyte mitochondria. Obviously the observed change of the OXPHOS complex organization is a characteristic feature for adipogenic hMSCs differentiation. By contrast, the prominent presence of higher oligomeric, possibly trimeric assembly forms of CV that can be observed in mitochondria of adipocytes seems not to be characteristic for adipogenic differentiation, as this assembly form also appears in the control upon longer cultivation of hMSCs.

### Differences in OXPHOS SC formation distinguishes adipocytes from hMSCs

**Figure 4:**
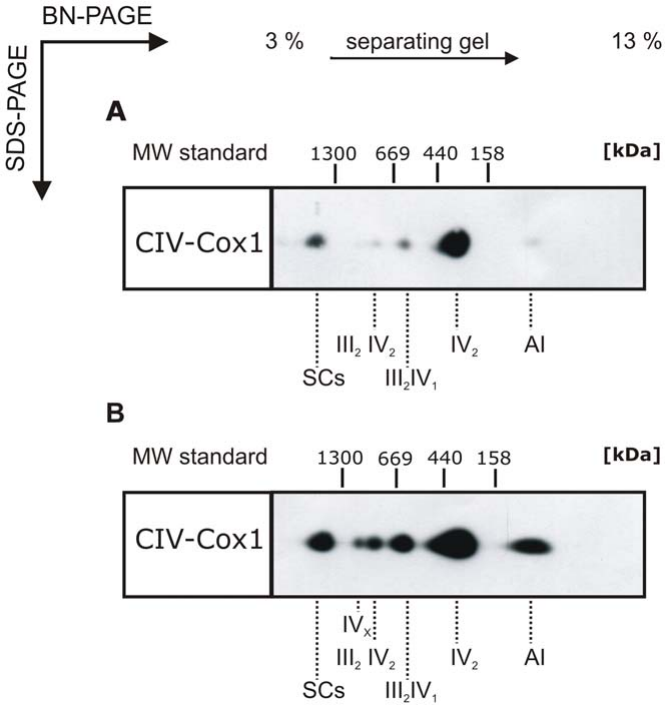
Supramolecular organization of cytochrome *c* oxidase. Identical amounts of mt proteins from undifferentiated hMSCs (A) and adipocytes (B) were run on a 2D-BN/SDS-PAGE, blotted and detected with antibody against subunit COX1. COX assembly forms and respective molecular weights are indicated according to Schägger and Pfeiffer (2000): supercomplexes (“SCs”), assembly intermediate (“AI”) and complex IV assembly forms (Roman numerals, x denotes an unknown number of CIV entities). Signal assigned as “SCs” comprises OXPHOS supercomplexes a, b, c and d according to Schägger and Pfeiffer (2000).

To address the question whether the enhancement of OXPHOS SC signals upon differentiation of hMSCs reflects a specific change in OXPHOS organization, Western blots of mt proteins separated by 2D-BN/SDS-PAGE, were analyzed with an antibody directed against subunit COX1. Semi-quantitative information about the differences in the abundance of SCs was obtained from Western blot analyses: signals obtained with adipocyte mitochondria reached saturation earlier than the ones obtained with MSC mitochondria. Comparison of Western blot images revealed stronger signals for SCs in mitochondria of adipocytes compared to stem cells (Figure 4B and 4A, respectively). A ∼10-fold longer exposure time is necessary to obtain comparable signals for SCs with mitochondria from hMSCs suggesting that the quantity of OXPHOS SCs is strikingly increased in adipocyte mitochondria. Additionally the supramolecular assembly forms III_2_IV_1_ and III_2_IV_2_ were less pronounced in the hMSCs mitochondria than in the adipocyte mitochondria. The assembly form IV_X_ (∼1.1 MDa) is not at all detectable and the COX assembly intermediate (AI) with a molecular weight of ∼150 kDa is almost undetectable in hMSCs mitochondria.

Clear differences during hMSCs differentiation both in the number and the intensity of signals reflecting the supramolecular organization of OXPHOS complexes could be confirmed in five independent experiments, and indicate that the absence or a relatively low amount of OXPHOS SCs may be a specific feature of undifferentiated hMSCs.

## Discussion

In this report we analyzed the mt phenotype of hMSCs during adipogenic differentiation. In accordance with a previous report focussing on osteogenic differentiation [2], we observed that differentiation of hMSCs is accompanied by a general increase in mt biogenesis.

Microscopical analyses show that adipocytes possess a characteristic distribution of the mt network. The typical perinuclear localization of hMSC mitochondria changes together with cell morphology upon induction of adipogenic differentiation. In line with the observations in ATSCs, human ESC lines and human HSCs (for review see [23]), our data support the hypothesis that perinuclear localization of mitochondria is a characteristic feature of stem cells or the mt phenotype of undifferentiated cells, respectively. *Vice versa*, the alteration in the distribution of the mt network indicates successful differentiation of hMSCs, and the simultaneous presence of both mt morphologies in a culture of differentiated cells hints at the heterogeneity of hMSCs. Mt morphology and dynamics were recently recognized as regulators of cellular processes such as proliferation and apoptosis [26]; consequently these features may also affect differentiation. Although the reason for such a specific mt localization is still obscure, this feature could possibly be exploited as a novel stemness marker [23].

As expected, upon differentiation of hMSCs the mRNA levels of marker proteins for hMSC (CD105 and CD 90) decreased, while the mRNA levels of adipogenic markers (adipsin, ADIPOQ, UCP1 and UCP2) increased. The increased expression of UCP1, actually an indicator for brown adipocyte differentiation, was unexpected. The mRNA level of ADIPOQ (adiponectin) which is a known marker for white adipocytes, was several fold upregulated (5.000×) compared to UCP1. These data indicate that either the differentiation of hMSCs according to standard protocols results in both brown and white adipocytes, or that UCP1 is also expressed during white adipocyte differentiation. The expression of ADIPOQ was also confirmed on the level of proteins using WB (data not shown). It was reported that brown and white adipocytes originate from different pools of mesenchymal precursors and that brown adipocytes are able to convert into adipocytes of the white phenotype when specific transcription factors are expressed [27].

The finding of elevated expression of PGC-1α clearly indicates mt biogenesis, and the increase in the abundance of mRNAs for other proteins necessary for oxidative metabolism reveals a metabolic switch during adipogenic differentiation of hMSCs. A similar increase of the respective genes was observed during osteogenic differentiation by Chen *et al.* [2] and in own experiments, indicating that mt biogenesis and the metabolic switch is not a lineage specific, but rather a general aspect of hMSC differentiation. It was shown previously that undifferentiated hMSCs produce high levels of lactate and consume less oxygen than their mature derivatives, suggesting dependence on glycolysis [2]. Generally, undifferentiated cells are usually in a quiescent state and mainly relying on anaerobic metabolism (for review see [28]). Whilst ATP produced by glycolysis is sufficient to supply the energy for the stem cell in their niche, OXPHOS is required to power differentiated cells. Consequently mt biogenesis and an adjustment of mt function during differentiation are necessary to create the energetic environment of mature cells. Mt biogenesis not only enlarges the mt network but also forms a specific mt phenotype as evident e.g. by changes in OXPHOS organization. Our 2D BN/SDS-PAGE analyses revealed clear differences in the supramolecular organization of the ETC. Additionally to a general rise in signal intensities of OXPHOS complexes and supramolecular assembly forms of CIV in adipocyte mitochondria we observed the appearance of assembly forms of CV subunits, indicating an ongoing OXPHOS formation and/or a high turn over of RC complexes. The most significant change in OXPHOS organization, however, was the increase in the abundance of OXPHOS SCs, most remarkably of the major form (CI CIII_2_ CIV). Based on the signal intensities it can be judged that adipocyte mitochondria contain strikingly more OXPHOS SCs than hMSCs.

During adipogenic differentiation additional supramolecular organization forms of CIV appeared: according to the literature these assembly forms were assigned as CIII_2_CIV and CIII_2_CIV_2_ [16]. In contrast, our data did not show an association of CIV_2_ with CIII_2_ suggesting alternative assembly partners, e.g. CV or CV subunits. The identity of the assembly partner of CIV_X_ (∼1.1 MDa) is also unknown, as none of the investigated complexes was associated with CIV at this MW form. Alternatively, the observed signal could originate from a not yet described oligomeric CIV assembly. The CIV signal with a molecular weight of 150 kDa (“AI”) could represent the COX assembly core consisting of a pre-assembled subcomplex comprising the COX subunits 1, 4 and 5a as described previously [21]. The formation of this subcomplex indicates an increased RC complex formation during adipogenic differentiation.

The organization of RC complexes in the form of SCs is thought to enable a more efficient transport of electrons leading to a higher OXPHOS activity. Recently it was shown for HSCs that mt respiration is able to modulate differentiation, but does not affect cell proliferation [29]. Accordingly, our data highlight the role of the mt phenotype and in particular the role of OXPHOS organization for adipogenic differentiation of hMSCs. The formation of RC SCs could be an essential prerequisite for the energy supply of mature cell types.

In summary, we show that adipogenic differentiation of hMSCs is accompanied by changes of the mt phenotype. Most remarkably the abundance of OXPHOS SCs, especially of the main form consisting of CI, CIII_2_ and CIV, is considerable lower in the ETC of hMSCs than in adipocytes. The observed changes in the OXPHOS complexes profile seem to be characteristic for the mt phenotype of adipogenic differentiating cells.

Even though hMSCs represent a relatively heterogeneous population cultivated for several days under normoxic conditions potentially bearing the risk of spontaneous differentiation as described for hESCs [7], we observed distinct changes in the OXPHOS profile during adipogenic differentiation. Possibly pure stem cells completely lack OXPHOS SCs, and the absence of SCs may serve as mt biomarker to assess stemness. Confirmation of this hypothesis requires further investigations analyzing highly purified stem cell populations cultivated under conditions similar to that of the stem cell niche.

Future studies comparing the OXPHOS organization of hMSCs and fibroblasts using 2D-BN/SDS-PAGE may also contribute to the ongoing debate about stem cell properties of hMSCs.
